# Acute kidney injury after major hepatectomy: association with postoperative complications and post-hepatectomy liver failure

**DOI:** 10.1093/bjsopen/zrag018

**Published:** 2026-03-24

**Authors:** Oskar Swartling, Tim Reese, Kristina Hasselgren, Jennie Engstrand, Anna Emilia Kern, Ruth Baumgartner, Poya Ghorbani, Per Sandström, Ernesto Sparrelid, Karl J Oldhafer, Bergthor Björnsson, Stefan Gilg

**Affiliations:** Division of Surgery and Oncology, Department of Clinical Sciences, Interventions and Technology, Karolinska Institutet, Stockholm, Sweden; Department of Upper Abdominal Diseases, Karolinska University Hospital, Stockholm, Sweden; Division of Surgery and Oncology, Department of Clinical Sciences, Interventions and Technology, Karolinska Institutet, Stockholm, Sweden; Department of Upper Abdominal Diseases, Karolinska University Hospital, Stockholm, Sweden; Department of Surgery and Clinical and Experimental Medicine, Linköping University, Linköping, Sweden; Division of Surgery and Oncology, Department of Clinical Sciences, Interventions and Technology, Karolinska Institutet, Stockholm, Sweden; Department of Upper Abdominal Diseases, Karolinska University Hospital, Stockholm, Sweden; Division of Surgery and Oncology, Department of Clinical Sciences, Interventions and Technology, Karolinska Institutet, Stockholm, Sweden; Department of Upper Abdominal Diseases, Karolinska University Hospital, Stockholm, Sweden; Department of Upper Abdominal Diseases, Karolinska University Hospital, Stockholm, Sweden; Division of Surgery and Oncology, Department of Clinical Sciences, Interventions and Technology, Karolinska Institutet, Stockholm, Sweden; Department of Upper Abdominal Diseases, Karolinska University Hospital, Stockholm, Sweden; Department of Surgery and Clinical and Experimental Medicine, Linköping University, Linköping, Sweden; Division of Surgery and Oncology, Department of Clinical Sciences, Interventions and Technology, Karolinska Institutet, Stockholm, Sweden; Department of Upper Abdominal Diseases, Karolinska University Hospital, Stockholm, Sweden; Division of Hepatobiliary and Pancreatic Surgery, Department of Surgery, Asklepios Hospital Barmbek, Hamburg, Germany; Department of Surgery and Clinical and Experimental Medicine, Linköping University, Linköping, Sweden; Division of Surgery and Oncology, Department of Clinical Sciences, Interventions and Technology, Karolinska Institutet, Stockholm, Sweden; Department of Upper Abdominal Diseases, Karolinska University Hospital, Stockholm, Sweden

**Keywords:** Renal injury, Liver surgery, Liver failure

## Abstract

**Background:**

Patients undergoing liver resections are at increased risk of acute kidney injury (AKI). The objective of this study was to examine AKI after major hepatectomy and the associated risk of adverse postoperative outcomes, including post-hepatectomy liver failure (PHLF) and mortality.

**Methods:**

All adult patients undergoing major hepatectomy between 2010 and 2021 at three tertiary referral centres in Sweden and Germany were included. Postoperative AKI was based on standardized criteria using serum creatinine and urine output. Logistic regression models were used to explore associations with adverse events.

**Results:**

In all, 1561 patients were included in the study, of whom 400 (25.6%) developed AKI of any grade. Risk factors for AKI included male sex, higher age, and a low preoperative estimated glomerular filtration rate. Patients with AKI were at increased risk of intensive care unit admission (adjusted odds ratio (aOR) 6.06; 95% confidence interval (c.i.) 2.83 to 12.95), Clavien–Dindo grade ≥ IIIa (aOR 2.08; 95% c.i. 1.61 to 2.67) and PHLF grade B or C (aOR 2.87; 95% c.i. 2.11 to 3.90). Patients with only AKI and patients with both AKI and PHLF grade B or C had an increased risk of 90-day mortality than patients with neither. Postoperative AKI was associated with an increased risk of 90-day mortality even after adjustment for PHLF.

**Conclusion:**

AKI after major hepatectomy is a common complication and is associated with considerable morbidity and mortality. Postoperative AKI may be both an early risk factor for PHLF after major hepatectomy and independently contribute to 90-day mortality.

## Teaser

This study explored early acute kidney injury after major hepatectomy and its association with post-hepatectomy liver failure. One-quarter of all included patients developed early acute kidney injury, and these patients were also more likely to later experience post-hepatectomy liver failure.

## Introduction

Acute kidney injury (AKI) is a common complication after gastrointestinal surgery with a prevalence of around 13%, with variations depending on the type of surgery and whether the surgery is elective or acute^1^. A sudden, temporary, or sustained decrease in kidney function during the perioperative period is associated with both co-morbidity and morbidity after surgery^2^, where also milder episodes can confer an increased risk of death^3^. Because a low estimated glomerular filtration rate (eGFR) increases the risk of postoperative AKI, and given the increase of chronic kidney disease (CKD) among the general population^4^, more patients undergoing surgery may be at risk of postoperative AKI. The most commonly used definition of AKI is the Kidney Disease: Improving Global Outcomes (KDIGO) guidelines^5^, defining AKI by changes in both serum creatinine and urine volume output, because oliguria with normal creatinine levels can be a presentation of AKI^6^. Patients undergoing major hepatectomy, including associating liver partition and portal vein ligation for staged hepatectomy (ALPPS), are at increased risk of postoperative AKI than patients undergoing minor liver resections^7–9^. It is assumed that, apart from general surgical risk factors such as high blood loss and long duration of surgery, there are also hepatectomy-specific risk factors for the development of AKI, including the hepatorenal syndrome^10^. A feared adverse event following major hepatectomy is post-hepatectomy liver failure (PHLF). AKI can be one feature of its presentation, although the extent to which immediate postoperative AKI contributes to the risk of developing PHLF or is a secondary effect of PHLF is unclear^11^. The aim of this study was to investigate the impact of postoperative AKI after major hepatectomy on patient outcome using standardized criteria for AKI based on both creatinine measurements and urine volume output. Furthermore, a possible association of AKI with other postoperative complications, including PHLF, was explored.

## Methods

### Study population

All patients aged ≥ 18 years who underwent major hepatectomy (defined as three or more liver segments) between 2010 and 2021 at three tertiary referral centres for hepatopancreatobiliary surgery (Karolinska University Hospital, Linköping University Hospital, both in Sweden, and Asklepios Hospital Hamburg, Germany) were included. In Asklepios Hospital, ALPPS remained standard, whereas in the remaining institutions it was only performed in research studies or as a rescue treatment after failed hypertrophy. Patient-related data were retrospectively collected from digital patient record systems. Patients without any postoperative measurements of creatinine or urine volume output were excluded.

The study was approved by regional ethics committees and the requirement for informed consent was waived due to the retrospective nature of the study.

### Exposures and measures

Postoperative AKI was defined using the KDIGO guidelines^5^. The guidelines define AKI as an increase in serum creatinine by ≥ 0.3 mg/dl (26.5 µmol/l) within 48 hours (h), or a ≥ 1.5-fold increase in serum creatinine from baseline within 7 days, or urine volume ≤ 0.5 ml/kg/h for 6 h^5^. In this study, to explore early AKI after major hepatectomy, only the first two postoperative days were used to define AKI. Further, because data on hourly urine output were not available, 24-h urine volume was used and AKI was defined as a urine volume of ≤ 0.5 ml/kg/h on either the first or second postoperative day. The grading of AKI (stage 1–3) was performed according to the KDIGO guidelines^5^, but stage 2 was defined as a urine output ≤ 0.5 ml/kg/h for 24 h on either the first or second postoperative day, and stage 3 was defined as a urine output ≤ 0.3 ml/kg/h for 24 h on either the first or second postoperative day. *Table S1* provides details on missing data for serum creatinine and urine output, as well as the timing of AKI diagnosis. Preoperative eGFR was based on the last recorded serum creatinine before surgery, which was routinely collected the day before surgery. Standardized future liver remnant (sFLR; *Table S2*) was calculated using preoperative information on the future liver remnant based on computed tomography, and total liver volume was estimated using the Vauthey formula^12^ based on body surface area according to the Mosteller formula^13^.

### Outcomes

The outcomes of interest included postoperative AKI, as defined above, postoperative complications, and mortality. Postoperative complications included Clavien–Dindo grade ≥ IIIa^14^, a hospital stay > 14 days, and readmission within 90 days. Biliary leakage and postoperative haemorrhage were defined according to the International Study Group of Liver Surgery (ISGLS), and grade B and C were considered clinically significant^15,16^. Intensive care unit (ICU) admission was standard for all operated patients at Asklepios Hospital and was therefore only evaluated in patients from centres without routine ICU admissions. PHLF was defined using the ISGLS criteria, with grades B and C considered clinically significant^17^. Time-to-event data for 90-day mortality are reported according to the presence of postoperative AKI and postoperative PHLF with or without postoperative AKI.

### Statistical analysis

Descriptive statistics for continuous variables are presented as either the mean and standard deviation (s.d.) or median and interquartile range (i.q.r.), with data analysed using Student’s *t* test or the Mann–Whitney *U* test. Categorical variables are presented as counts and percentages and were analysed using the χ^2^ test or Fischer’s exact test. To investigate intra- and postoperative risk factors for AKI after surgery, an analysis was performed using unadjusted and adjusted logistic regression models presenting odds ratios (ORs) with their 95% confidence interval, (c.i.). Multivariable models were adjusted for the predefined preoperative variables. AKI and postoperative complications were also analysed using unadjusted and adjusted logistic regression models with the adjusted models including the same variables. Univariable and multivariable Cox proportional hazards models, assessed with the Schoenfeld residuals test, were used for time-to-event data to assess 90-day mortality. Additional adjustment was performed for PHLF when the impact of AKI alone was assessed. Kaplan–Meier estimates of survival were used to visualize 90-day mortality. Analyses were performed using Stata^®^ version 16.1 software (StataCorp, College Station, TX, USA). Two-sided *P* < 0.05 was considered statistically significant.

## Results

### Study population

A total of 1573 patients underwent hepatectomy during the study period; 12 did not have postoperative measurements of creatinine or urine output allowing for AKI categorization and so were excluded. The remaining 1561 patients were included in the study. The median age at the time of surgery was 65 (i.q.r. 56–73) years and most (882 patients, 56.5%) were male (*Table 1*). As indicated in *Table 2*, the most common indication for surgery was colorectal liver metastases (742, 47.5%), followed by cholangiocarcinoma (295, 18.9%), hepatocellular carcinoma (188, 12.0%), and gallbladder cancer (31, 2%), whereas the most common procedure was right hemihepatectomy (736, 47.1%), followed by extended right hemihepatectomy (307, 19.7%), left hemihepatectomy (309, 19.8%), extended left hemihepatectomy (111, 7.1%), and ALPPS step 2 (98, 6%). All included procedures were open major hepatectomies. In total, 617 of all included patients (39.5%) received neoadjuvant oncological treatment (*Table 1*). The median follow-up time was 1.9 (i.q.r. 0.7–4.1) years.

**Table 1:** Patient characteristics overall and according to the presence of postoperative AKI

	Postoperative AKI			P*
	No AKI (*n* = 1161)	AKI (*n* = 400)	Overall (*n* = 1561)	
**Baseline characteristics**				
Age (years), median (i.q.r.)	65 (55–72)	67 (57–73)	65 (56–73)	0.043
Missing	6 (0.5%)	0 (0%)	6 (0.4%)	
BMI (kg/m^2^), mean(s.d.)	25.1(4.3)	27.6(4.8)	25.7(4.5)	< 0.001
Missing	5 (0.4%)	2 (0.5%)	7 (0.5%)	
Sex				< 0.001
Female	558 (48.1%)	121 (30.3%)	679 (44.5%)	
Male	603 (51.9%)	279 (69.7%)	882 (56.5%)	
Preoperative creatinine† (µmol/l), mean(s.d.)	74.1(21.1)	81.9(27.7)	76.1(23.3)	< 0.001
Missing	44 (4%)	8 (2%)	52 (3%)	
Preoperative eGFR (ml/min/1.73 m^2^), mean(s.d.)	86.5(18.9)	82.6(22.2)	85.5(19.9)	< 0.001
Missing	50 (4%)	8 (2%)	58 (4%)	
Preoperative eGFR categories				< 0.001
≥90 ml/min/1.73 m^2^	506 (45.3%)	157 (40.0%)	663 (43.9%)	
≥60 to < 90 ml/min/1.73 m^2^	509 (45.6%)	172 (44.9%)	681 (45.1%)	
≥45 to < 60 ml/min/1.73 m^2^	75 (7%)	38 (10%)	113 (7.5%)	
< 45 ml/min/1.73 m^2^	27 (2%)	25 (6%)	52 (3%)	
Co-morbidities				
Cardiovascular disease	440 (37.9%)	233 (58.3%)	673 (43.1%)	< 0.001
Missing	1 (0.1%)	0 (0%)	1 (0.06%)	
Diabetes	136 (11.7%)	79 (20%)	215 (13.8%)	< 0.001
Missing	1 (0.1%)	0 (0%)	1 (0.06%)	
Pulmonary disease	94 (8%)	34 (9%)	128 (8.2%)	0.807
Missing	2 (0.2%)	0 (0%)	2 (0.1%)	
**Disease characteristics**				
Liver disease				
Fibrosis	546 (65.9%)	152 (67.9%)	698 (66.3%)	0.002
Cirrhosis	22 (2%)	14 (4%)	36 (2%)	0.065
Missing	333 (28.7%)	176 (44.0%)	509 (32.6%)	
Diagnosis				< 0.001
CRLM	580 (50.0%)	162 (40.5%)	742 (47.6%)	
CCC	193 (16.7%)	102 (25.6%)	295 (18.9%)	
HCC	130 (11.2%)	58 (15%)	188 (12.1%)	
Gallbladder cancer	21 (2%)	10 (3%)	31 (2%)	
Other	235 (20.3%)	68 (17%)	303 (19.4%)	
Missing	2 (0.2%)	0 (0%)	2 (0.1%)	
**Treatment or interventions**				
Neoadjuvant treatment	489 (42.4%)	128 (32.3%)	617 (39.8%)	< 0.001
Missing	7 (0.6%)	4 (1%)	11 (0.7%)	
Portal vein embolization or ligation	197 (17.2%)	103 (26.3%)	300 (19.5%)	< 0.001
Missing	16 (1.4%)	9 (2.3%)	25 (1.6%)	
Pringle manoeuvre	214 (18.4%)	88 (22%)	302 (19.4%)	0.114
Time of Pringle manoeuvre (min), mean(s.d.)	24.4(12.3)	26.4(13.9)	24.9(12.7)	0.338

Values are *n* (%) unless otherwise stated. The presence of postoperative AKI is based on definitions suggested by KDIGO. †Creatinine (mg/dL) = creatinine (µmol/L) ÷ 88.4. AKI, acute kidney injury; i.q.r., interquartile range; BMI, body mass index; s.d., standard deviation; eGFR, estimated glomerular filtration rate; CRLM, colorectal liver metastases; CCC, cholangiocarcinoma; HCC, hepatocellular carcinoma. *Analysed using Student‘s t-test or Mann-Whitney U-test for continous variables and chi-square or Fischer‘s exact test for categorical variables.

**Table 2:** ORs for AKI according to perioperative characteristics

Perioperative characteristic	No. of individuals (%)	No. of patients with AKI (%)	ORs for developing AKI*			
			Unadjusted	*P*	Adjusted†	*P*
**Type of resection**						
Right hemihepatectomy	736 (47.2%)	182 (24.7%)	Reference		Reference	
Left hemihepatectomy	309 (19.8%)	68 (22%)	0.86 (0.63, 1.18)	0.347	0.84 (0.60, 1.18)	0.324
Extended right	307 (19.7%)	88 (29%)	1.22 (0.91, 1.65)	0.186	1.34 (0.97, 1.85)	0.072
Extended left	111 (7.1%)	21 (19%)	0.71 (0.43, 1.18)	0.183	0.68 (0.40, 1.15)	0.148
ALPPS Step 2	98 (6%)	41 (42%)	2.19 (1.42, 3.38)	< 0.001	2.55 (1.55, 4.20)	< 0.001
Operation duration ≥ 4 h	997 (63.9%)	262 (26.3%)	1.10 (0.87, 1.40)	0.431	0.95 (0.74, 1.23)	0.716
Pringle manoeuvre	302 (19.4%)	88 (29%)	1.25 (0.95, 1.66)	0.115	1.16 (0.86, 1.56)	0.337
Blood loss (≥ 1000 ml)	1047 (67.1%)	314 (29.9%)	2.13 (1.63, 2.78)	< 0.001	1.90 (1.44, 2.53)	< 0.001
sFLR	271 (17.3%)	64 (24%)				
< 30%	98 (6%)	26 (10%)	Reference		Reference	
30 to < 40%	99 (6%)	19 (7%)	0.66 (0.34, 1.29)	0.222	0.74 (0.36, 1.51)	0.406
≥ 40%	74 (5%)	19 (7%)	0.96 (0.48, 1.90)	0.899	1.00 (0.47, 2.12)	1.000

*Values in parentheses are 95% confidence intervals. †Adjusted for sex, age at the time of surgery, preoperative estimated glomerular filtration rate, diabetes, cardiovascular disease, pulmonary disease, and body mass index. ORs, odds ratios; AKI, acute kidney injury; ALPPS, associated liver partition with portal vein ligation for staged hepatectomy; sFLR, standardized future liver remnant.

### Postoperative AKI after major hepatectomy

When applying the KDIGO criteria for AKI using creatinine and urine volume measurements on the first two postoperative days, 400 of all patients who underwent major hepatectomy (25.6%) developed AKI of any stage. AKI stage 1 was diagnosed in 130 (8.3%) patients, 199 (12.7%) patients had AKI stage 2, and 71 (5%) patients had AKI stage 3. The majority of all patients who developed AKI after surgery (352, 88.0%) fulfilled the KDIGO criteria within the first postoperative day. Patients with higher age, male sex, higher body mass index, higher preoperative creatinine levels, cardiovascular disease, and diabetes were more likely to develop postoperative AKI (*Table 1*). Among patients who received neoadjuvant oncological treatment, which is only administered to patients with colorectal liver metastases, 20.7% (128) developed AKI. The surgical risk factors for postoperative AKI included undergoing an ALPPS step 2 procedure and intraoperative blood loss ≥ 1000 ml (adjusted OR 2.55 (95% c.i. 1.55 to 4.20) and 1.90 (95% c.i. 1.44 to 2.53), respectively; *Table 2*). There were no differences in sFLR and the odds of AKI (for example, for sFLR ≥ 40 *versus* < 30%: adjusted OR 1.00; 95% c.i. 0.47 to 2.12).

### AKI and the risk of postoperative adverse outcomes

Patients with AKI after major hepatectomy also had an increased risk of other postoperative complications. Among the 1044 patients not admitted to the ICU routinely after surgery, patients with AKI had an increased odds of ICU admission within the first day after surgery (adjusted OR 6.06; 95% c.i. 2.83–12.95; *Table 3*). In addition, patients with AKI had an increased odds of readmission within 90 days (adjusted OR 1.67; 95% c.i. 1.11 to 2.52), a hospital stay ≥ 14 days (adjusted OR 2.47; 95% c.i. 1.91 to 3.18), and Clavien–Dindo grade ≥ IIIa complications (adjusted OR 2.08; 95% c.i. 1.61 to 2.67). Postoperative AKI was associated with postoperative haemorrhage grade ≥ B (adjusted OR 2.07; 95% c.i. 1.14 to 3.77). Of all included patients, 245 developed PHLF grade ≥ B, and 108 of these patients also developed postoperative AKI (*Table 3*). Patients with AKI also had an increased odds of PHLF grade B or C compared with patients with normal postoperative kidney function (adjusted OR 2.87; 95% c.i. 2.11 to 3.90).

**Table 3:** Postoperative AKI and ORs for postoperative complications after major hepatectomy

Postoperative complication	No. of individuals (%)	No. of patients with AKI (%)	Postoperative AKI *versus* no AKI*			
			Unadjusted OR	*P*	Adjusted OR†	*P*
ICU admission within 24 h‡	37 (4%)	23 (62%)	6.92 (3.50, 13.70)	< 0.001	6.06 (2.83, 12.95)	< 0.001
Readmission within 90 days	176 (19.1%)	46 (26%)	1.56 (1.06, 2.28)	0.024	1.67 (1.11, 2.52)	0.014
Hospital stay ≥ 14 days	503 (32.5%)	187 (37.2%)	2.40 (1.89, 3.04)	< 0.001	2.47 (1.91, 3.18)	< 0.001
Clavien–Dindo grade ≥ IIIa	519 (33.9%)	180 (34.7%)	2.01 (1.58, 2.54)	< 0.001	2.08 (1.61, 2.67)	< 0.001
PHLF grade B or C	245 (15.8%)	108 (44.1%)	2.77 (2.09, 3.68)	< 0.001	2.87 (2.11, 3.90)	< 0.001
Biliary leakage grade ≥ B	169 (11.3%)	53 (31%)	1.37 (0.97, 1.94)	0.078	1.37 (0.94, 1.99)	0.096
Postoperative haemorrhage grade ≥ B	56 (4%)	23 (41%)	2.08 (1.20, 3.58)	0.009	2.07 (1.14, 3.77)	0.017

*Values in parentheses are 95% confidence intervals. Unadjusted and adjusted ORs from logistic regression analysis are shown for postoperative complications after major hepatectomy comparing postoperative AKI according to Kidney Disease: Improving Global Outcomes to normal postoperative kidney function. †Adjusted for sex, age at the time of surgery, preoperative estimated glomerular filtration rate, diabetes, cardiovascular disease, pulmonary disease, and body mass index. ‡Among patients not routinely admitted to the ICU after surgery (*n* = 1044). AKI, acute kidney injury; ORs, odds ratios; ICU, intensive care unit; h, hours; PHLF, post-hepatectomy liver failure.

### Postoperative mortality

In all, 89 patients died before the 90-day follow-up. Compared with patients with normal postoperative kidney function, patients with postoperative AKI had an increased risk of 90-day mortality (adjusted hazard ratio (HR) 3.70; 95% c.i. 2.33 to 5.88; *Table 4* and *Fig. S1*). This risk remained increased for all AKI stages (stage 1: adjusted HR 4.08 (95% c.i. 2.26 to 7.37); stage 2: adjusted HR 2.76 (95% c.i. 1.50 to 5.07); stage 3: adjusted HR 5.53 (95% c.i. 2.81 to 10.87). After additionally adjusting for PHLF grade B or C, patients with postoperative AKI still had an increased risk of 90-day mortality (HR 2.37; 95% c.i. 1.49 to 3.79; *Table 4*). The HRs comparing patients with postoperative AKI, PHLF, or the combination of AKI and PHLF are presented in *Table 5*. Compared with patients without AKI or PHLF, patients with AKI alone had an increased risk of 90-day mortality (adjusted HR 2.28; 95% c.i. 1.13 to 4.60), but patients with PHLF alone had a higher risk of 90-day mortality (adjusted HR 6.33; 95% c.i. 3.27 to 12.26; *Fig. S2*). Patients with AKI and PHLF after surgery had an even higher risk of 90-day mortality (adjusted HR 16.64; 95% c.i. 9.33 to 29.67). The risk for adverse postoperative outcomes among patients who developed AKI, including PHLF and 90-day mortality, were similar after excluding people who underwent the ALPPS step 2 procedure (*Table S3* and *Fig. S3*). Among patients who developed PHLF, a more severe AKI stage was associated with increased mortality (*Table S4*).

**Table 4:** Cox’s proportional hazards regression analysis for 90-day mortality after major hepatectomy according to postoperative AKI and AKI stage

	Unadjusted HR	*P*	Adjusted HR*	*P*	Adjusted HR†	*P*
Postoperative AKI	3.42 (2.25, 5.19)	< 0.001	3.70 (2.33, 5.88)	< 0.001	2.37 (1.49, 3.79)	< 0.001
**AKI stage**						
No AKI	Reference		Reference		Reference	
Stage 1	4.09 (2.36, 7.10)	< 0.001	4.08 (2.26, 7.37)	< 0.001	2.81 (1.55, 5.11)	0.001
Stage 2	2.37 (1.33, 4.21)	< 0.001	2.76 (1.50, 5.07)	0.001	1.67 (0.91, 3.10)	0.101
Stage 3	5.21 (2.74, 9.98)	< 0.001	5.53 (2.81, 10.87)	< 0.001	3.49 (1.77, 6.90)	< 0.001

Values in parentheses are 95% confidence intervals. Unadjusted and adjusted HRs are shown for postoperative mortality according to AKI and AKI stage. *Adjusted for sex, age at the time of surgery, preoperative estimated glomerular filtration rate, diabetes, cardiovascular disease, pulmonary disease, and body mass index. †Additionally adjusted for post-hepatectomy liver failure grade B or C. AKI, acute kidney injury; HR, hazard ratio.

**Table 5:** Cox’s proportional hazards regression analysis for 90-day mortality after major hepatectomy according to postoperative AKI and PHLF grade B or C

	Unadjusted HR	*P*	Adjusted HR*	*P*
No AKI or PHLF	Reference		Reference	
AKI	2.27 (1.17, 4.41)	0.016	2.28 (1.13, 4.60)	0.022
PHLF	6.40 (3.49, 11.76)	< 0.001	6.33 (3.27, 12.26)	< 0.001
AKI and PHLF	15.07 (8.81, 25.77)	< 0.001	16.64 (9.33, 29.67)	< 0.001

Values in parentheses are 95% confidence intervals. Unadjusted and adjusted HRs are shown for postoperative mortality according to the presence of AKI. *Adjusted for sex, age at the time of surgery, preoperative estimated glomerular filtration rate, diabetes, cardiovascular disease, pulmonary disease, and body mass index. AKI, acute kidney injury; PHLF, post-hepatectomy liver failure; HR, hazard ratio.

## Discussion

In this study of 1561 patients undergoing major hepatectomy at three tertiary referral centres, postoperative AKI of any grade, based on serum creatinine and urine output, was prevalent (26% of all operated patients). Pre- and intraoperative risk factors for AKI included male sex, higher age, lower preoperative eGFR, ALPPS step 2 procedure, and higher intraoperative blood loss. Patients with postoperative AKI were more likely to develop PHLF, whereas patients with both postoperative AKI alone and in combination with PHLF had a significantly higher 90-day mortality.

It has previously been shown that patients undergoing any type of liver resection are at risk of developing AKI after surgery, with an incidence ranging from 9 to 25% depending on the degree of liver resection^8,9,18–20^. The reasons for a comparatively high risk of kidney injury after liver resection remain uncertain, although factors such as intraoperative hypotension, acute tubular necrosis, or haemodynamic changes in portal blood flow may increase the susceptibility of the kidneys during the immediate operative period^10,21^. In addition, the definition of AKI in general has varied and only recently been harmonized following the introduction of the KDIGO criteria^5,22,23^. In the present study on major hepatectomies, a direct relationship between sFLR and the risk of developing AKI was not found, suggesting that other factors contribute to postoperative AKI rather than a reduction in liver volume alone. It should be noted that only a minority of included patients were evaluated for sFLR. Current treatment practices at the three institutions in the present study include the measurement of central venous pressure, aimed at low pressure (< 5 mmHg) during surgery, which is thought to decrease perioperative blood loss^24,25^ PHLF is a major cause of short-term mortality following liver surgery^26–28^, with an incidence of around 10% after major hepatectomy^29^. In the present study, postoperative AKI was an independent risk factor for PHLF and, in combination with AKI, significantly contributed to postoperative mortality. It has previously been shown that an increase in portal vein pressure to the remnant liver after hepatectomy can increase the risk of AKI^30^. The mechanism is hypothesized to involve a hepatorenal reflex that causes kidney hypoperfusion^10,31,32^. At the same time, liver failure in itself can cause AKI through the hepatorenal syndrome. Based on the current ISGLS definition of PHLF, which does not allow a diagnosis of PHLF earlier than 5 days after surgery, these results suggest that postoperative AKI within the first 2 days after hepatectomy may be an early sign of adverse postoperative events in general, and PHLF in particular. However, it is not possible by this study alone to clearly define the cause or the sequence of postoperative AKI and PHLF, and pathophysiological changes probably interact in the immediate postoperative period. AKI is likely to be caused by a number of interrelated factors associated with the patient, the procedure, or the postoperative course that are not directly due to the to the future liver remnant. However, the results of this study suggest that meeting the definition of AKI early after surgery is associated with a higher risk of developing PHLF, by the current definitons of AKI and PHLF. Therefore early AKI in the postoperative phase could be considered as a high-risk sign of an adverse outcome^33^.

This study has limitations. It was impossible to distinguish between AKI due to the actual liver resection or postoperative kidney failure caused by emerging PHLF. A decrease in urine output after major surgery could also be affected by factors other than direct kidney injury. In addition, the results may have been influenced by the practices at the three institutions, and sFLR was only measured in a minority of patients included in the study, which could cause selection bias. Another limitation is that information on changes in eGFR after ALPPS step 1 was not available. The 2-day cut-off for AKI after surgery could miss some cases of AKI, although this cut-off was chosen to better reflect direct postoperative causes of AKI. The study did not include information on how AKI was treated during the postoperative course with regard to, for example, initiation of dialysis, fluid resuscitation, and the duration of abnormal creatinine levels, although this may not invalidate the mere presence of AKI being associated with PHLF.

This study of patients undergoing major hepatectomy found that the prevalence of postoperative AKI was unexpectedly high and that it was associated with other postoperative complications. Postoperative AKI within 2 days after surgery was as independent risk factor for PHLF grade B and C. Patients with AKI alone or in combination with PHLF had a significantly increased risk of 90-day mortality, and AKI was an independent risk factor for mortality also after adjusting for PHLF.

## Supplementary Material

zrag018_Supplementary_Data

## Data Availability

Data sets generated during and/or analysed during the present study are not publicly available but are available from the corresponding author upon reasonable request.

## References

[1] STARSurg Collaborative . Impact of postoperative acute kidney injury in patients undergoing major gastrointestinal surgery on 1-year survival and renal outcomes: a national multicentre cohort study. BJS Open 2021;5. doi: 10.1093/bjsopen/zrab134 PMC8759520 35029656

[2] Hoste EAJ, Kellum JA, Selby NM, Zarbock A, Palevsky PM, Bagshaw SM et al Global epidemiology and outcomes of acute kidney injury. Nat Rev Nephrol 2018;14:607–625 30135570 10.1038/s41581-018-0052-0

[3] Bihorac A, Yavas S, Subbiah S, Hobson CE, Schold JD, Gabrielli A et al Long-term risk of mortality and acute kidney injury during hospitalization after major surgery. Ann Surg 2009;249:851–858 19387314 10.1097/SLA.0b013e3181a40a0b

[4] Prowle JR, Croal B, Abbott TEF, Cuthbertson BH, Wijeysundera DN; METS Study Investigators. Cystatin C or creatinine for pre-operative assessment of kidney function and risk of post-operative acute kidney injury: a secondary analysis of the METS cohort study. Clin Kidney J 2024;17:sfae004 38269033 10.1093/ckj/sfae004 PMC10807905

[5] Khwaja A . KDIGO clinical practice guidelines for acute kidney injury. Nephron Clin Pract 2012;120:c179–c184 22890468 10.1159/000339789

[6] Kellum JA, Sileanu FE, Murugan R, Lucko N, Shaw AD, Clermont G. Classifying AKI by urine output *versus* serum creatinine level. J Am Soc Nephrol 2015;26:2231–2238 25568178 10.1681/ASN.2014070724 PMC4552117

[7] Bredt LC, Peres LAB. Risk factors for acute kidney injury after partial hepatectomy. World J Hepatol 2017;9:815–822 28706580 10.4254/wjh.v9.i18.815 PMC5491404

[8] Kambakamba P, Slankamenac K, Tschuor C, Kron P, Wirsching A, Maurer K et al Epidural analgesia and perioperative kidney function after major liver resection. Br J Surg 2015;102:805–812 25877255 10.1002/bjs.9810

[9] Reese T, Kroger F, Makridis G, Drexler R, Jusufi M, Schneider M et al Impact of acute kidney injury after extended liver resections. HPB (Oxford) 2021;23:1000–1007 33191106 10.1016/j.hpb.2020.10.015

[10] Peres LA, Bredt LC, Cipriani RF. Acute renal injury after partial hepatectomy. World J Hepatol 2016;8:891–901 27478539 10.4254/wjh.v8.i21.891 PMC4958699

[11] Soreide JA, Deshpande R. Post hepatectomy liver failure (PHLF)—recent advances in prevention and clinical management. Eur J Surg Oncol 2021;47:216–224 32943278 10.1016/j.ejso.2020.09.001

[12] Vauthey JN, Abdalla EK, Doherty DA, Gertsch P, Fenstermacher MJ, Loyer EM et al Body surface area and body weight predict total liver volume in Western adults. Liver Transpl 2002;8:233–240 11910568 10.1053/jlts.2002.31654

[13] Mosteller RD . Simplified calculation of body-surface area. N Engl J Med 1987;317:1098 3657876 10.1056/NEJM198710223171717

[14] Dindo D, Demartines N, Clavien PA. Classification of surgical complications: a new proposal with evaluation in a cohort of 6336 patients and results of a survey. Ann Surg 2004;240:205–213 15273542 10.1097/01.sla.0000133083.54934.ae PMC1360123

[15] Koch M, Garden OJ, Padbury R, Rahbari NN, Adam R, Capussotti L et al Bile leakage after hepatobiliary and pancreatic surgery: a definition and grading of severity by the International Study Group of Liver Surgery. Surgery 2011;149:680–688 21316725 10.1016/j.surg.2010.12.002

[16] Rahbari NN, Garden OJ, Padbury R, Maddern G, Koch M, Hugh TJ et al Post-hepatectomy haemorrhage: a definition and grading by the International Study Group of Liver Surgery (ISGLS). HPB (Oxford) 2011;13:528–535 21762295 10.1111/j.1477-2574.2011.00319.x PMC3163274

[17] Rahbari NN, Garden OJ, Padbury R, Brooke-Smith M, Crawford M, Adam R et al Posthepatectomy liver failure: a definition and grading by the International Study Group of Liver Surgery (ISGLS). Surgery 2011;149:713–724 21236455 10.1016/j.surg.2010.10.001

[18] Slankamenac K, Beck-Schimmer B, Breitenstein S, Puhan MA, Clavien PA. Novel prediction score including pre- and intraoperative parameters best predicts acute kidney injury after liver surgery. World J Surg 2013;37:2618–2628 23959337 10.1007/s00268-013-2159-6

[19] Dedinska I, Mikolajcik P, Skalova P, Mokan M, Laca L. Acute kidney injury after liver resection in elderly patients. BMC Nephrol 2019;20:272 31319808 10.1186/s12882-019-1449-0 PMC6639960

[20] Ishikawa S, Tanaka M, Maruyama F, Fukagawa A, Shiota N, Matsumura S et al Effects of acute kidney injury after liver resection on long-term outcomes. Korean J Anesthesiol 2017;70:527–534 29046772 10.4097/kjae.2017.70.5.527 PMC5645585

[21] Liao P, Zhao S, Lyu L, Yi X, Ji X, Sun J et al Association of intraoperative hypotension with acute kidney injury after liver resection surgery: an observational cohort study. BMC Nephrol 2020;21:456 33138788 10.1186/s12882-020-02109-9 PMC7607844

[22] Bellomo R, Ronco C, Kellum JA, Mehta RL, Palevsky P; Acute Dialysis Quality Initiative Workgroup. Acute renal failure—definition, outcome measures, animal models, fluid therapy and information technology needs: the second international consensus conference of the Acute Dialysis Quality Initiative (ADQI) group. Crit Care 2004;8:R204–R212 10.1186/cc2872 PMC522841 15312219

[23] Mehta RL, Kellum JA, Shah SV, Molitoris BA, Ronco C, Warnock DG et al Acute Kidney Injury Network: report of an initiative to improve outcomes in acute kidney injury. Crit Care 2007;11:R31 17331245 10.1186/cc5713 PMC2206446

[24] Hughes MJ, Ventham NT, Harrison EM, Wigmore SJ. Central venous pressure and liver resection: a systematic review and meta-analysis. HPB (Oxford) 2015;17:863–871 26292655 10.1111/hpb.12462 PMC4571753

[25] Stephanos M, Stewart CMB, Mahmood A, Brown C, Hajibandeh S, Hajibandeh S et al Low *versus* standard central venous pressure during laparoscopic liver resection: a systematic review, meta-analysis and trial sequential analysis. Ann Hepatobiliary Pancreat Surg 2024;28:115–124 38361339 10.14701/ahbps.23-137 PMC11128796

[26] Gilg S, Sandstrom P, Rizell M, Lindell G, Ardnor B, Stromberg C et al The impact of post-hepatectomy liver failure on mortality: a population-based study. Scand J Gastroenterol 2018;53:1335–1339 30345846 10.1080/00365521.2018.1501604

[27] Jara M, Reese T, Malinowski M, Valle E, Seehofer D, Puhl G et al Reductions in post-hepatectomy liver failure and related mortality after implementation of the LiMAx algorithm in preoperative work-up: a single-centre analysis of 1170 hepatectomies of one or more segments. HPB (Oxford) 2015;17:651–658 26058324 10.1111/hpb.12424 PMC4474514

[28] Baumgartner R, Gilg S, Bjornsson B, Hasselgren K, Ghorbani P, Sauter C et al Impact of post-hepatectomy liver failure on morbidity and short- and long-term survival after major hepatectomy. BJS Open 2022;6:zrac097 35849062 10.1093/bjsopen/zrac097 PMC9291378

[29] Vitello DJ, Shah D, Ko B, Brajcich BC, Peters XD, Merkow RP et al Establishing the clinical relevance of grade A post-hepatectomy liver failure. J Surg Oncol 2024;129:745–753 38225867 10.1002/jso.27570 PMC10922784

[30] Reiterer C, Taschner A, Luf F, Hecking M, Tamandl D, Zotti O et al Effect of liver resection-induced increases in hepatic venous pressure gradient on development of postoperative acute kidney injury. BMC Nephrol 2022;23:21 34996372 10.1186/s12882-021-02658-7 PMC8742325

[31] Moreau R, Lebrec D. Acute kidney injury: new concepts. Hepatorenal syndrome: the role of vasopressors. Nephron Physiol 2008;109:p73–p79 18802378 10.1159/000142939

[32] Sparrelid E, Olthof PB, Dasari BVM, Erdmann JI, Santol J, Starlinger P et al Current evidence on posthepatectomy liver failure: comprehensive review. BJS Open 2022;6. doi: 10.1093/bjsopen/zrac142 PMC9681670 36415029

[33] Baumgartner R, Engstrand J, Rajala P, Grip J, Ghorbani P, Sparrelid E et al Comparing the accuracy of prediction models to detect clinically relevant post-hepatectomy liver failure early after major hepatectomy. BJS 2024;111. doi: 10.1093/bjs/znad433 PMC10763542 38150185

