# Supplementary material for: Acute kidney injury after major hepatectomy: association with postoperative complications and post-hepatectomy liver failure
Source: BJS Open. 2026 Mar 24;10(2):zrag018. doi: 10.1093/bjsopen/zrag018 (PMC13010065; doi:10.1093/bjsopen/zrag018)
Supplement: zrag018_Supplementary_Data [file zrag018_supplementary_data.docx]

**Acute Kidney Injury after Major Hepatectomy: Association with Postoperative Complications and Post-Hepatectomy Liver Failure**

*Oskar Swartling^1,2^, Tim Reese^1,2^, Kristina Hasselgren^3^, Jennie Engstrand^1,2^, Anna Emilia Kern^1,2^, Ruth Baumgartner^2^, Poya Ghorbani^1,2^, Per Sandström^3^, Ernesto Sparrelid^1,2^, Karl J. Oldhafer^4^, Bergthor Björnsson^3^, Stefan Gilg^1,2^*

^1^Division of Surgery and Oncology, Department of Clinical Sciences, Interventions and Technology, Karolinska Institutet, Stockholm, Sweden

^2^Department of Upper Abdominal Diseases, Karolinska University Hospital, Stockholm, Sweden

^3^Department of Surgery and Clinical and Experimental Medicine, Linköping University, Sweden.

^4^Division of Hepatobiliary and Pancreatic Surgery, Department of Surgery, Asklepios Hospital Barmbek, Hamburg, Germany

**Corresponding author.** Oskar Swartling. [oskar.swartling@ki.se](mailto:oskar.swartling@ki.se). **ORCID ID**; 0000-0002-3783-1567

**Supplementary Materials - Index**

| **Supplementary Figures and Tables** |  |
| --- | --- |
| **Table S1.** Criteria and missing values for acute kidney injury after major hepatectomy | *page 3* |
| **Table S2.** Included patients by presence of postoperative acute kidney injury and sFLR | *page 4* |
| **Table S3.** Postoperative acute kidney injury and odds ratios for postoperative complications after major hepatectomy, excluding people undergoing ALPPS Step 2 procedure | *page 5* |
| **Table S4.** Hazard ratio of 90-day mortality after major hepatectomy by postoperative acute kidney injury stage among people with post-hepatectomy liver failure grade B or C. | *page 6* |
| **Figure S1.** Kaplan-Meier estimate of 90-day survival by postoperative acute kidney injury (AKI). | *Page 7* |
| **Figure S2.** Kaplan-Meier estimate of 90-day survival by acute kidney injury (AKI) and post-hepatectomy liver failure (PHLF). | *Page 8* |
| **Figure S3.** Kaplan-Meier estimate of 90-day survival by postoperative acute kidney injury (AKI) and excluding people who underwent ALPPS Step 2 procedure. | *page 9* |

**Supplementary Methods**

Patient characteristics included sex, age at the time of surgery, preoperative body mass index (BMI) and preoperative creatinine. Preoperative eGFR was calculated using the 2009 Chronic Kidney Disease Epidemiology Collaboration equation without correction for race (1). Other baseline characteristics included presence of cardiovascular disease, diabetes and pulmonary disease (all binary) and underlying histopathological diagnosis was categorized as colorectal liver metastasis (CRLM), cholangiocarcinoma (CCC), hepatocellular carcinoma (HCC), gallbladder cancer (GBC) and other/miscellaneous. Fibrosis and cirrhosis were defined by the pathology report of non-cancerous liver parenchyma.

**Supplementary Figures and Tables**

| **Table S1.** Criteria for acute kidney injury after major hepatectomy | | |
| --- | --- | --- |
| **Criteria** | **Patients fulfilling criteria**  **for acute kidney injury**  ***n* (%)** | **Available measurements** |
| Increase in serum creatinine by ≥0.3 mg/dl (26.5 µmol/l) within 48 hours, n (%) | 216 (13.8%) | 1543 (99%) |
| Day 1 | 156 (9.9%) | 1471 (94%) |
| Day 2 | 162 (10.4%) | 1478 (95%) |
| Increase in serum creatinine to ≥1.5 times preoperative creatinine level within 7 days after surgery, n (%) | 137 (8.8%) | 1556 (99%) |
| Day 1 | 141 (9.0%) | 1515 (97%) |
| Day 2 | 142 (9.1%) | 1480 (95%) |
| Urine volume <0.5 ml/kg/h for 24 hours 1 or 2 days after surgery, n (%) | 229 (14.7%) | 1210 (78%) |
| Day 1 | 86 (5.5%) | 1050 (67%) |
| Day 2 | 163 (10.4%) | 1137 (74%) |

| **Table S2.** Included patients by presence of postoperative acute kidney injury and sFLR | | | | | | | |
| --- | --- | --- | --- | --- | --- | --- | --- |
|  | **Postoperative AKI** | | | |  | |  |
| **Variables** | **No AKI** | | **AKI** | | **Overall** | |  |
| sFLR, *n* (%) |  |  |  |  |  |  |  |
| < 30% | 72 | (35%) | 26 | (41%) | 98 | (36%) |  |
| 30 to < 40% | 80 | (39%) | 19 | (30%) | 99 | (37%) |  |
| ≥ 40% | 55 | (27%) | 19 | (30%) | 74 | (27%) |  |
| Missing, *n* (%) | 954 | (82%) | 336 | (84%) | 1290 | (83%) |  |
| Abbreviations: sFLR, standardized future liver remnant; AKI, acute kidney injury. | | | | | | | |

| **Table S3.** | Postoperative acute kidney injury and odds ratios for postoperative complications after major hepatectomy, excluding people undergoing ALPPS Step 2 procedure | | | | | | | | | | | |
| --- | --- | --- | --- | --- | --- | --- | --- | --- | --- | --- | --- | --- |
|  | |  | |  | | **Postoperative AKI vs. no AKI** | | | | | | |
| **Postoperative complication** | | **No. of individuals** | | **Proportion with AKI** | | **Unadjusted OR**  (95% CI) | | **P-value** |  | **Adjusted OR**  (95% CI) | | **P-value** |
| ICU admission within 24 hours^1^ | | 36 | (4%) | 22 | (61%) | 6.59 | (3.31–13.13) | <0.001 |  | 5.81 | (2.69–12.54) | <0.001 |
| Readmission within 90 days | | 170 | (10%) | 45 | (26%) | 1.60 | (1.09–2.36) | 0.018 |  | 1.70 | (1.12–2.57) | 0.012 |
| Hospital stay ≥ 14 days | | 462 | (32%) | 164 | (36%) | 2.31 | (1.80–2.95) | <0.001 |  | 2.35 | (1.80–3.07) | <0.001 |
| Clavien-Dindo grade ≥IIIa | | 479 | (33%) | 163 | (35%) | 2.09 | (1.64–2.68) | <0.001 |  | 2.12 | (1.63–2.76) | <0.001 |
| PHLF grade B or C | | 227 | (16%) | 97 | (43%) | 2.79 | (2.07–3.75) | <0.001 |  | 2.82 | (2.04–3.88) | <0.001 |
| Biliary leakage grade ≥ B | | 156 | (11%) | 49 | (31%) | 1.46 | (1.02–2.10) | 0.039 |  | 1.41 | (0.96–2.09) | 0.079 |
| Postoperative haemorrhage grade ≥ B | | 52 | (4%) | 21 | (40%) | 2.14 | (1.21–3.77) | 0.009 |  | 2.36 | (1.27–4.38) | 0.006 |
| Unadjusted and adjusted logistic regression of postoperative complications after major hepatectomy comparing postoperative acute kidney injury according to KDIGO to normal postoperative kidney function. Adjusted for sex, age at the time of surgery, preoperative estimated glomerular filtration rate, diabetes, cardiovascular disease, pulmonary disease and body mass index.  ^1^Among patients not routinely admitted to the ICU postoperatively (n=1,044).  Abbreviations: AKI, acute kidney injury; OR, odds ratio; ICU, intensive care unit; PHLF, post-hepatectomy liver failure; ALPPS, Associating Liver Partition and Portal Vein Ligation for Staged Hepatectomy. | | | | | | | | | | | | |

| **Table S4.** | Hazard ratio of 90-day mortality after major hepatectomy by postoperative acute kidney injury stage among people with post-hepatectomy liver failure grade B or C | | | | | | | |
| --- | --- | --- | --- | --- | --- | --- | --- | --- |
|  | | **Unadjusted HR**  (95% CI) | | **P-value** |  | **Adjusted HR^1^**  (95% CI) | | **P-value** |
| **People with PHLF^2^** | |  | |  |  |  | |  |
| No AKI | | Reference | |  |  | Reference | |  |
| AKI Stage 1 | | 2.52 | (1.20–5.20) | 0.015 |  | 2.48 | (1.11–5.34) | 0.027 |
| AKI Stage 2 | | 1.71 | (0.83–3.52) | 0.146 |  | 1.55 | (0.71–3.38) | 0.272 |
| AKI Stage 3 | | 3.97 | (1.79–8.79) | 0.001 |  | 3.23 | (1.38–7.60) | 0.007 |
| Unadjusted and adjusted hazard ratios for postoperative mortality by AKI stage. Analysed with Cox’s proportional hazards regression model.  ^1^Adjusted for sex, age at the time of surgery, preoperative eGFR, diabetes, cardiovascular disease, pulmonary disease and BMI.  ^2^Number of patients = 245  Abbreviations: AKI, acute kidney injury; PHLF, post-hepatectomy liver failure; HR, hazard ratio. | | | | | | | | |


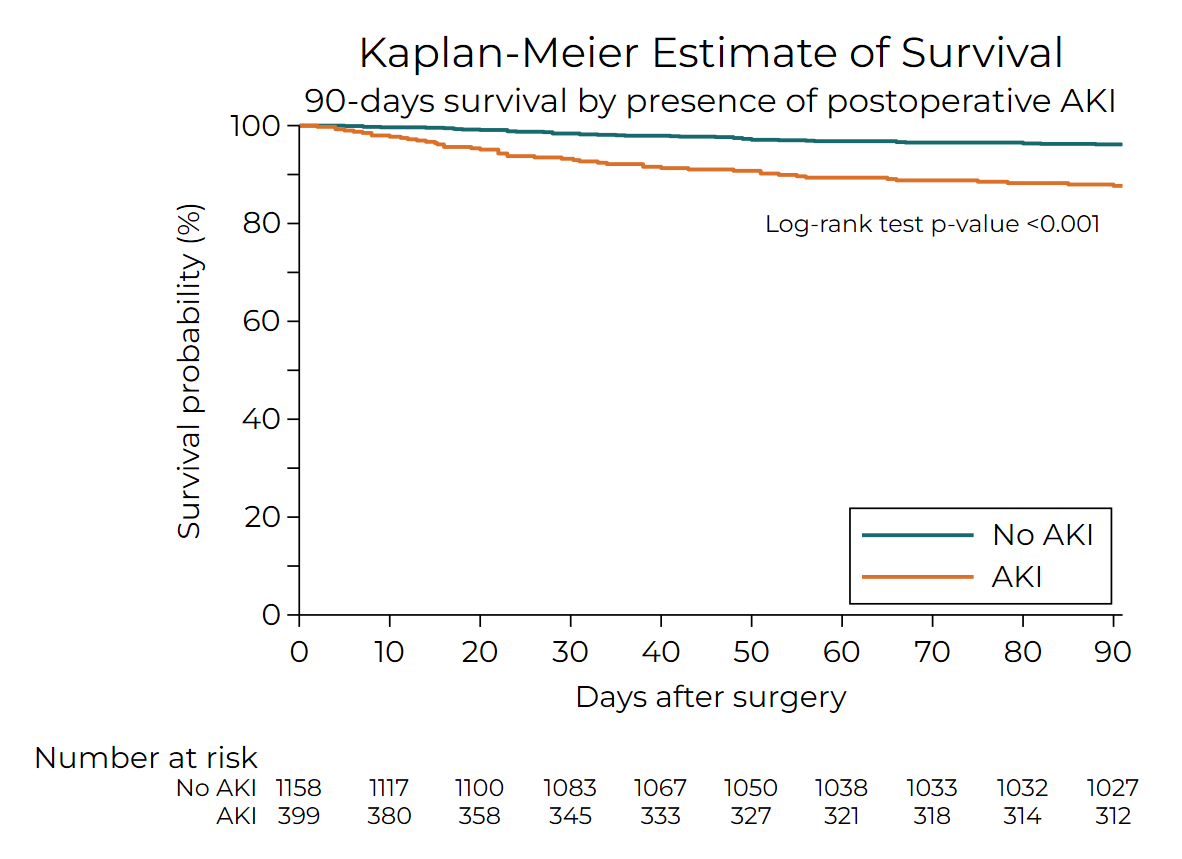


**Figure S1.** Kaplan-Meier estimate of 90-day survival by postoperative acute kidney injury (AKI).


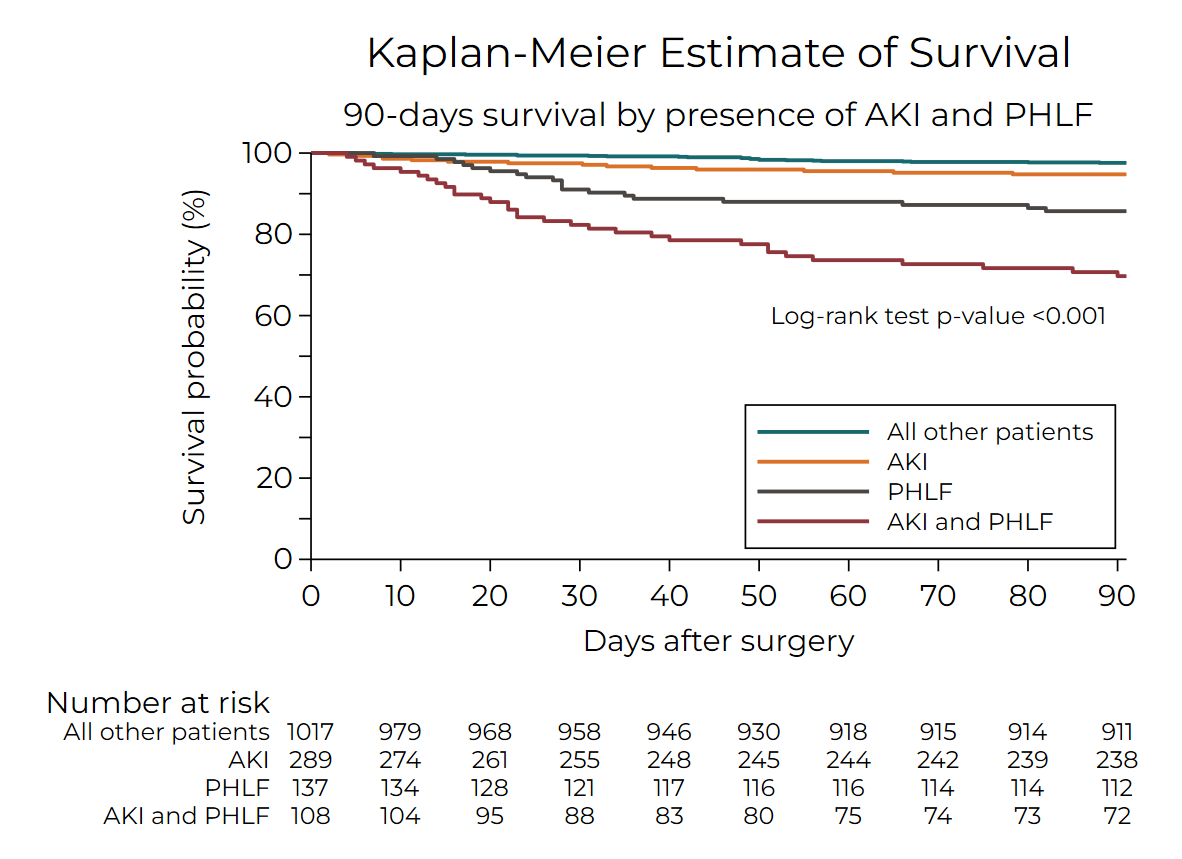


**Figure S2.** Kaplan-Meier estimate of 90-day survival by acute kidney injury (AKI) and post-hepatectomy liver failure (PHLF).


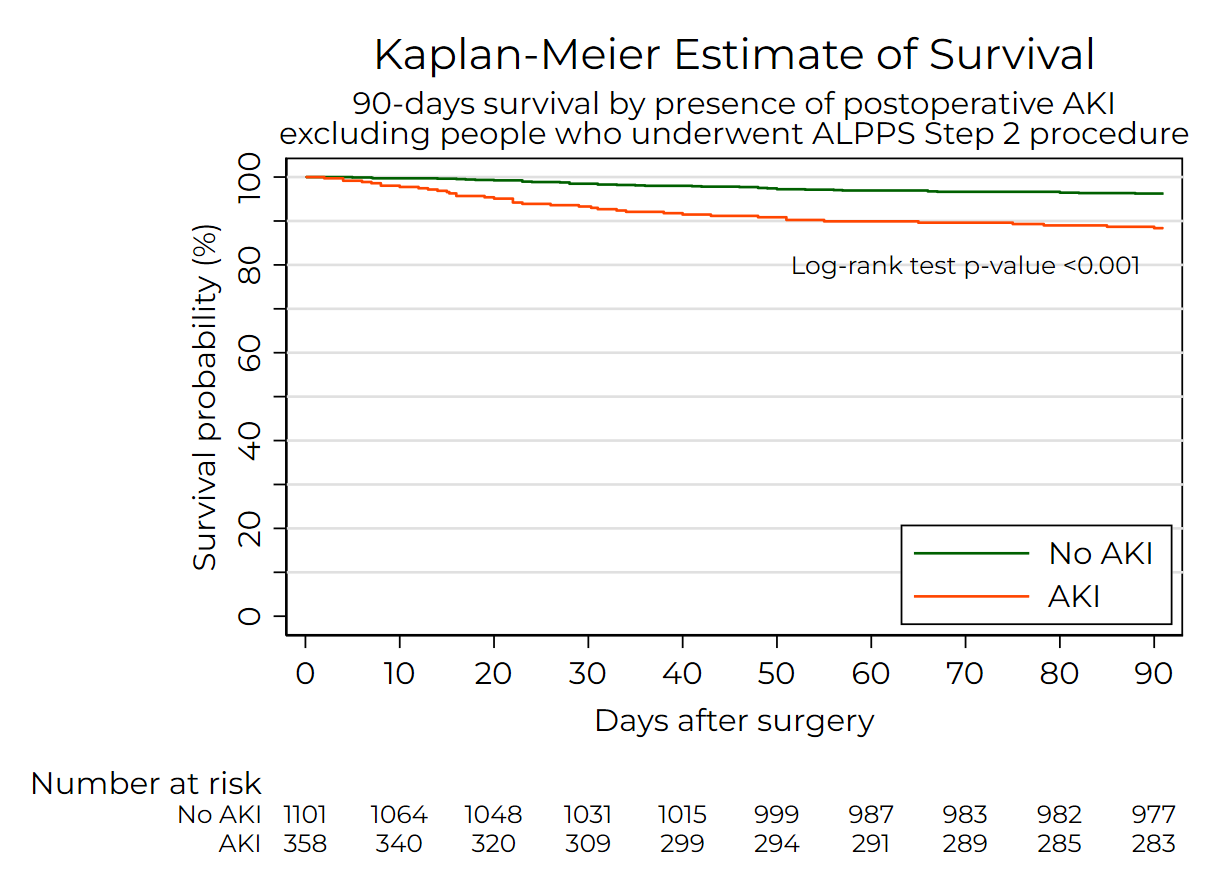


**Figure S3.** Kaplan-Meier estimate of 90-day survival by postoperative acute kidney injury (AKI) and excluding people who underwent ALPPS Step 2 procedure.

**References**

1.Levey AS, Stevens LA, Schmid CH, Zhang YL, Castro AF, Feldman HI, et al. A new equation to estimate glomerular filtration rate. Ann Intern Med. 2009;150(9):604-12.
